# Ultrastructural Changes in the Tegument and Tissues of *Fasciola hepatica* Adults and Their Eggs Due to the Effect of an Ethyl Acetate Extract of *Artemisia ludoviciana* Nutt. spp *Mexicana*

**DOI:** 10.1007/s11686-024-00933-9

**Published:** 2024-10-25

**Authors:** Alonso Ezeta-Miranda, José Guillermo Avila-Acevedo, Adriana Montserrat Espinosa-González, José del Carmen Benítez-Flores, Gerardo Francisco-Marquez, Yolanda Vera-Montenegro

**Affiliations:** 1 Laboratorio de Fitoquímica, UBIPRO, FES-Iztacala, UNAM. Av. de los Barrios # 1, Tlalnepantla Estado de México, 54010 México; 2 Laboratorio de Histología, UMF, FES-Iztacala, UNAM. Av. de los Barrios # 1, Tlalnepantla Estado de México, 54010 México; 3 Facultad de Medicina Veterinaria y Zootecnia, Departamento de Parasitología, UNAM, México, CDMX, México City, 04510 México

**Keywords:** Artemisia ludoviciana, Extract, Fasciolosis, Histology, Microscopy

## Abstract

**Purpose:**

The objective of the present work was to evaluate the effect of an ethyl acetate extract of *Artemisia ludoviciana* on the viability of adult *Fasciola hepatica* parasites and eggs.

**Methods:**

The collection of plant material was performed as described in previous reports. The dried material was macerated with ethyl acetate. Ovicidal assays were performed at concentrations of 100, 200, 300, 400 and 500 mg/L *A. ludoviciana* extract. Bioassays of fasciolicidal efficacy in adult specimens of *F. hepatica* were performed at extract concentrations of 125, 250, 375 and 500 mg/L. The effects of triclabendazole, a reference drug, and artemisinin were also evaluated.

**Results:**

The ovicidal effectiveness of the extracts obtained after 16 h of incubation at concentrations of 100, 200, 300, 400 and 500 mg/L was 48%, 52%, 87%, 89% and 92%, respectively (*p* < 0.05), and the fasciolicidal efficiencies during the first 24 h post-treatment ranged from 82 to 100% (*p* < 0.05). In both cases, scanning electron microscopy revealed damage to the shells of the eggs treated with the extract, compromising their stability. In adult fasciolae, alterations to the integument that resulted in its erosion and detachment were observed. Histopathological studies of the affected specimens revealed damage to the tegumentary and subtegumentary cells and alterations in the ovaries, testicles and intestine. This damage was more severe after treatment with the extract than after treatment with the other compounds.

**Methods:**

Extract of *A. ludoviciana* causes damage to the tegument, intestine, ovaries, testes and eggs of *F. hepatica*.

## Highlights

•Evaluate the effect of an ethyl acetate extract of *Artemisia ludoviciana* on the viability of adult *Fasciola hepatica* parasites and eggs.
•The ovicidal effectiveness of the extracts obtained after 16 h of incubation at concentrations of 100, 200, 300, 400 and 500 mg/L was 48%, 52%, 87%, 89% and 92%, respectively (p<0.05), and the fasciolicidal efficiencies during the first 24 h post-treatment ranged from 82% to 100% (p<0.05).
•Scanning electron microscopy revealed damage to the shells of the eggs treated with the extract, compromising their stability.
•In parasites exposed to extract exhibited partial erosion of the integument in different regions, damage to the tegument that affected the integrity of the spines was seen. In some cases, severe damage to the muscles was observed.
•Histological evaluation observe areas of advanced deterioration and regions. Alterations at this level allow substances access to deep organs, which can then be damaged. In this study, extract affected virtually the entire interior of the organism, including the digestive tract, the gonads, the internal parenchyma, and the subepidermal cells.

**Graphical Abstract:**

## Graphical abstract

**Figure:**
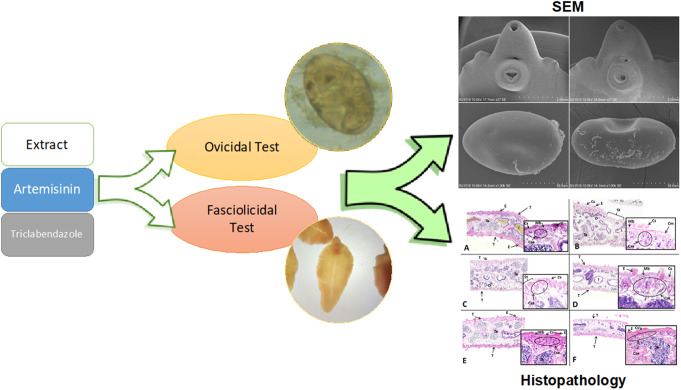
The objective of the present work was to evaluate the effect of an ethyl acetate extract of *Artemisia ludoviciana* on the viability of adult *Fasciola hepatica* parasites and eggs. In both cases, scanning electron microscopy revealed damage to the shells of the eggs treated with the extract. In adult fasciolae, alterations to the integument that resulted in its erosion and detachment were observed. Histopathological studies of the affected specimens revealed damage to the tegumentary and subtegumentary cells and alterations in the ovaries, testicles and intestine.

## Introduction

*Fasciola hepatica* is a trematode that lives in the bile ducts of mammals in general and requires snails of the genus *Lymnaea* spp to complete its larval cycle. The effects of this trematode on livestock are a decrease in productive parameters, illnesses, and, in some cases, death, and these effects result in an annual loss of millions of dollars for farms [1, 2]. Fasciolosis is a zoonosis that has an impact on public health; it affects 61 countries, and an estimated 17 million people are affected worldwide and 180 million are at risk of infection [3–6].

Historically, fascioliasis has been treated with various compounds; the most used is triclabendazole, which acts against both the young and the adult forms of the parasite [3]. With the passage of time and due to the widespread use of drugs, erroneous diagnoses, the expansion of livestock farms, adaptation and distribution of intermediate hosts due to climate change, and genetic diversity and adaptation within *F. hepatica*, there have been cases of anthelmintic resistance in several regions in Europe, Oceania and South America [7–19]. This has motivated us to explore new methods for treating the parasite safely. One such method involves the use of plants with anthelmintic effects and a search for new molecules derived from their secondary metabolites. One of these plants, *Artemisia ludoviciana* Nutt. spp *mexicana*, has been shown to possess in vitro efficacy against young adults and eggs *of F. hepatica* [20, 21]. Presumably, its fasciolicidal effect is due to the artemisinin and others sesquiterpen lactones present in extracts of the plant [22]. Previously, Ezeta and collaborators (2024) [21] evaluated the ovicidal percentage and the fasciolicidal efficacy of the extract and the objective of the present work was to evaluate the effect of an ethyl acetate extract of *Artemisia ludoviciana* on the tegument and tissues of adult *F. hepatica* parasites and eggs.

## Methodology

### Plant Material and Extract Procedures of *A. Ludoviciana* Nutt. spp. *Mexicana*

The collection of plant material was performed as described in previous reports [20, 22, 23] in the vicinity of the Teaching, Research and Extension Center in Tropical Livestock of the Faculty of Veterinary Medicine and Zootechnics (FMVZ) of the National Autonomous University of Mexico (UNAM), located in Martínez de la Torre, municipality of Tlapacoyan, Veracruz, Mexico (19°57′42″ N, 97°12′39″ W). Healthy leaves of *A. ludoviciana* Nutt. spp. mexicana were collected, and the taxonomic identification was performed in the herbarium of the Faculty of Higher Studies, Iztacala, UNAM (FESI-UNAM), and number 2156-IZTA was assigned. The green matter was dried at a constant temperature of 60 °C for three days and subsequently ground. The material was cold-macerated with ethyl acetate (EtAc). The extract was filtered and concentrated to dryness under reduced pressure in a Heidolph^®^ Mod. A Laborota 4000 rotary evaporator. The dried extract was stored at 4 °C in the dark. The EtAc extract obtained from *A. ludoviciana* (EAEAL) was used to perform the biological tests described in this study. A thin-layer chromatography was performed to corroborate the metabolites present in this new extract and ensure the same chromatographic pattern previously described by Ezeta et al. (2020) [22].

### Evaluation of the Ovicidal Activity of *A. ludoviciana* Ethyl Acetate Extract (EAEAL) and Fixation of Eggs for Scanning Electron Microscopy (SEM)

*F. hepatica* eggs were collected directly from the gallbladders of sheep affected by fasciolosis at the municipal slaughterhouse of Toluca, State of Mexico. The livers were maintained at a temperature of 4 to 8 °C during transport to the laboratory. The bile was obtained aseptically from the livers and mixed with 500 ml of distilled water (DH_2_O), after which the mixture was allowed to stand for approximately 20 to 30 min to allow the eggs to settle. After this period, 2/3 of the volume of the mixture was decanted, and the portion containing the eggs was brought to its original volume again with DH_2_O and allowed to rest for the same amount of time. This process was continued until the liquid was clear, and the eggs were then recovered. The eggs were kept at 4 °C for 24 h before ovicidal evaluation [14, 24, 25]. To carry out the ovicidal assay, 24-well NUNC© boxes were used, and 90 to 110 *F. hepatica* eggs were placed in each well. The eggs were exposed in triplicate to EAEAL at concentrations of 100, 200, 300, 400 and 500 mg/L; the eggs in the control wells received EtAc at 0.1 ml (equivalent to the maximum permissible amount for reconstituting the extract prior to the stock solution) and no treatment. The ovicidal activity of artemisinin (ART) obtained from SIGMA-ALDRICH©(USA), 98% purity (CAS Number: 63968-64-9) at concentrations of 10 and 20 mg/L was also evaluated. The boxes containing the eggs were incubated for 16 days at 28 °C and 80% humidity. After this period, they were exposed to artificial light for 2 h to allow the miracidia to hatch. Ovicidal activity was evaluated according to the following formula [26–29]:

$$Eggs\,hatched\left( \% \right) = \frac{{number\,of\,eggs\,hatched}}{{total\,number\,of\,eggs}} \times 100 $$

$$Ovicidal{\mkern 1mu} activity\left( \% \right) = \frac{\begin{gathered}\% {\mkern 1mu} eggs{\mkern 1mu} hatched{\mkern 1mu} in{\mkern 1mu} control - \hfill \\\% {\mkern 1mu} eggs{\mkern 1mu} hatched{\mkern 1mu} after{\mkern 1mu} drug{\mkern 1mu} incubation \hfill \\ \end{gathered} }{{\% {\mkern 1mu} eggs{\mkern 1mu} hatched{\mkern 1mu} in{\mkern 1mu} control}} \times 100$$

To observe the effects of EAEAL at 100 and 500 mg/L, the eggs were placed on Millipore filters, washed 2 to 3 times with DH_2_O and immersed in 4% formalin for 24 h; they were then washed again with DH_2_O and dehydrated in an ascending series of ethanol. They were subsequently dried to their critical point with extra-dry carbon dioxide (CO_2_), fixed on aluminum bases with carbon adhesive strips, and metallized with a 20-mA gold film for 2 min [26]. The samples were observed under a 10–15 kW S450 Hitachi scanning electron microscope within the facilities of the UNAM Institute of Biology (IB-UNAM).

### Collection of Adult *Fasciola hepatica* Parasites and in vitro Evaluation of the EAEAL

*F. hepatica* adults were obtained directly from the livers of infected cattle at the municipal slaughterhouse of Toluca, State of Mexico, Mexico. The specimens were washed with phosphate-buffered saline (PBS) to remove excess blood and bile. They were then placed in Roswell Park Memorial Institute (RPMI)-1640 medium HIMEDIA© (India) Lot number: 0000532004, at 37 °C and transported to the Experimental Helminth Chemotherapy Laboratory of the Department of Parasitology of the FMVZ-UNAM. At the laboratory, the parasites were washed several times with RPMI-1640 medium at 37 °C and placed in 20-ml dishes in appropriate culture medium (50% RPMI-1640 medium + 50% bovine serum) with 4 flukes per box (ratio of 1 fluke per 5 ml of medium). A mixture of antibiotics (100 IU penicillin + 100 mg/ml streptomycin) was added to the culture medium to prevent bacterial growth [21].

For the in vitro evaluation, a stock solution of EAEAL at a concentration of 500 mg/L was prepared; from this, dilutions were made to obtain the tested concentrations. Adult fasciolae were exposed in triplicate to EAEAL at 125, 250, 375 and 500 mg/L, incorporating the corresponding controls for EtAc to confirm that the solvent did not affect the parasite. Additionally negative controls without treatment were used. Triclabendazole (TCBZ) at a concentration of 10 mg/L used as a reference drug; at the same time, the response of ART at a concentration of 10 mg/L was evaluated. The boxes were incubated at 37 °C in a 5% CO_2_ atmosphere, and readings were taken at 24 and 48 h postexposure [30–36]. To evaluate the fasciolicidal efficacy of EAEAL and that of the drugs, the mobility and mortality criteria described by Huson et al. (2021) [37] and Jeyathilakan et al. (2010) [38] were considered. All procedures were performed under aseptic conditions using a BG© Mod. CFLV-130 laminar flow hood [20, 22, 33].

The fasciolicidal efficacy of EAEAL was evaluated, and the survival of the specimens in the treated group was compared to that of those in the control group [39].

$$Efficacy\left( \% \right) = \frac{\begin{gathered} Number\,of\,live\,flukes\,in\,the\,control\,group - \hfill \\ Number\,of\,lives\,flukes\,in\,the\,treated\,group \hfill \\ \end{gathered} }{{Number\,of\,live\,flukes\,in\,the\,control\,group}} \times 100$$

### Fixation and Observation of Adult Specimens of *F. hepatica* by SEM

The damage caused to the parasite by exposure to EAEAL at concentrations of 125 and 500 mg/L, exposure to ART at 10 mg/L and exposure to TCBZ at 10 mg/L was observed by SEM; untreated specimens were also examined for comparison. The adult fasciolae specimens were washed with 9% physiological saline solution (SSF) and preserved in 2% formalin for 24 h. They were then washed again with SSF and placed in AFA (water-alcohol-formaldehyde-acetic acid) fixative for 24 h at a temperature of 4 °C. At the end of that time, they were dehydrated in an ascending series of ethanol and dried to their critical point with extra-dry CO_2_. When dry, they were fixed onto carbon adhesive strips, metallized, and observed under a 10–15 kW S450 Hitachi scanning electron microscope at the IB-UNAM facilities, as described previously for eggs of this species [40–42].

### Histological Preparation of Adult Specimens of *F. hepatica*

The specimens were fixed in 4% paraformaldehyde for 48 h and then embedded in paraffin using the conventional method. Each specimen was transversely sectioned into three parts to obtain cross-sections at three levels of the body. Sections 4–5 microns in thickness were prepared from each individual and stained using the conventional hematoxylin-eosin (H-E) method. The histological sections were observed under a Leica DM 500 microscope and photographed using the Leica EZ program [43, 44].

### Analysis of Data

The observed ovicidal and fasciolicidal efficiencies (given as percentages) were analyzed using analysis of variance (ANOVA) and probit analysis with a 95% confidence interval using SYSTAT v.13.2 (Grafiti LLC. USA, 2024) to identify significant differences among the groups that received different treatments.

## Results

### Extract Performance

The initial amount of fresh plant matter collected was 3.07 kg. A total of 201.22 g of dry plant was obtained, and the total amount of dry EAEAL was 15.2 g. The total yield of dry matter was 7.55%.

### Ovicidal Activity of EAEAL

In all ovicidal tests, no alterations were observed in the untreated controls and the EtAc controls.

**Fig. 1:**
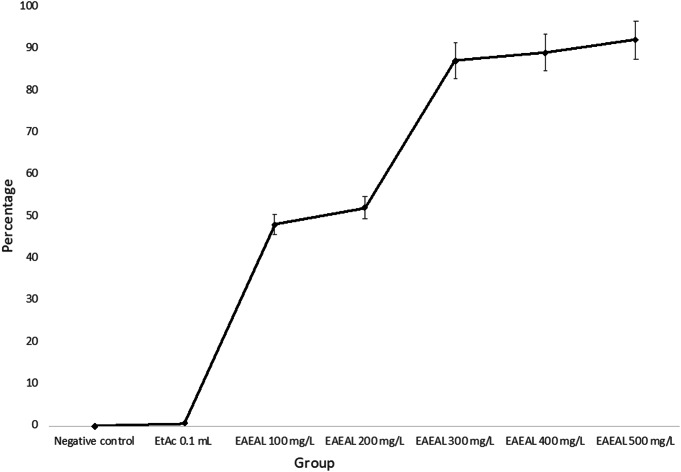
Ovicidal percentages of the different evaluation groups

The ovicidal efficiencies (given as percentages) of EAEAL at concentrations of 100, 200, 300, 400 and 500 mg/L were 48%, 52%, 87%, 89% and 92%, respectively (*P* < 0.05) (Fig. 1), obtaining percentages similar to those described by Ezeta et al. (2024).

**Fig. 2:**
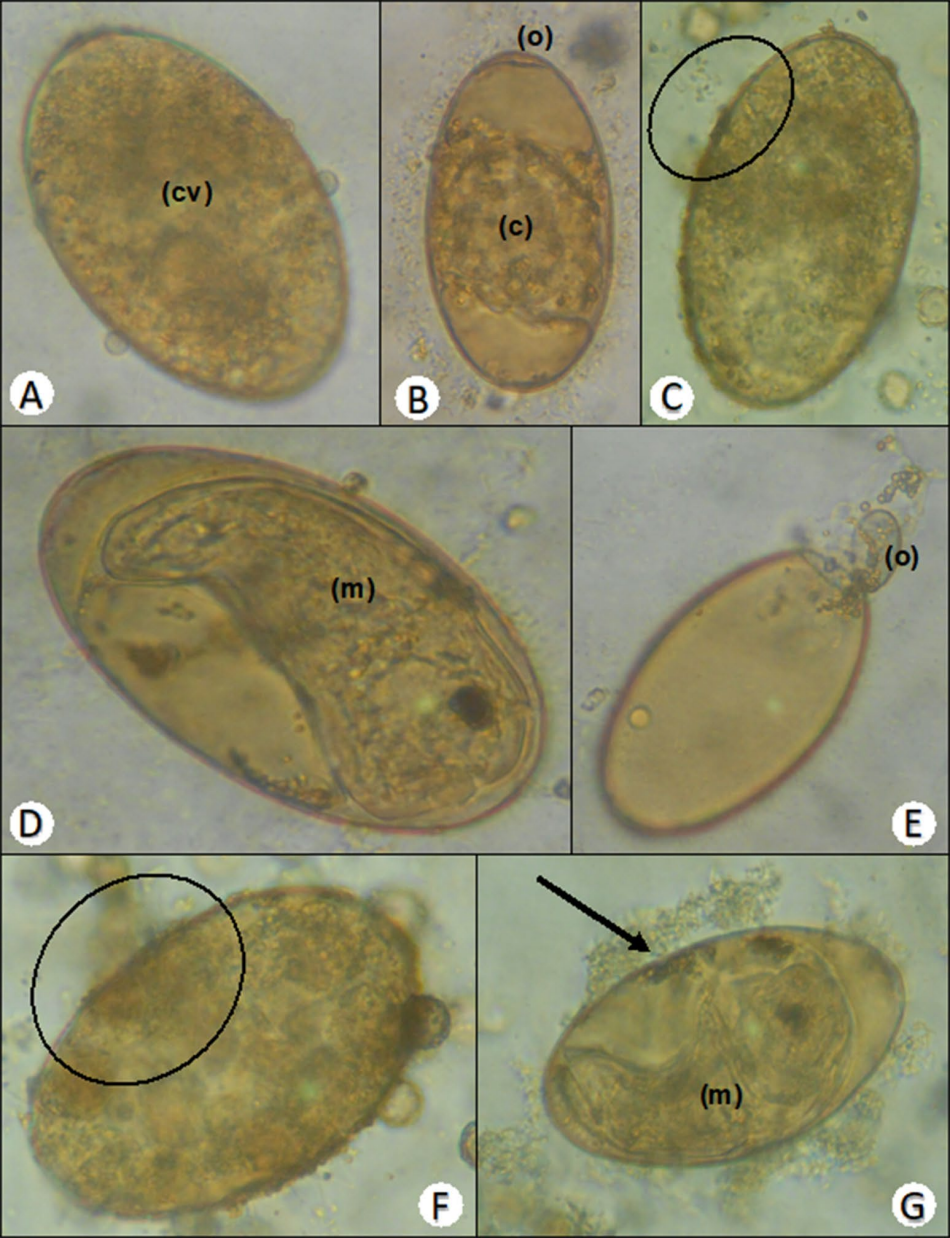
Appearance of F. hepatica eggs during the EAEAL ovicidal test. (**A**) Untreated egg on Day 1 of the test; the zygote and yolk (cv) can be seen. (**B**) Untreated egg on Day 7 of incubation. Zygote formation is observed (c), and the operculum can be seen (o). (**C**) Egg treated with 500 mg/L EAEAL; on Day 7 of incubation, an altered region can be seen on the periphery of the shell (circle). (**D**) Untreated egg on Day 16 of incubation, before exposure to light, showing complete formation of the miracidia (m). (**E**) Empty egg after photoexposure. (**F**) After the eggs were treated with 500 mg/L EAEAL for 16 days, damage to the shell and apparent leakage of yolk content were observed (circle). (**G**) Egg treated with 500 mg/L EAEAL on Day 16 of incubation, showing the development of miracidia (m) and leakage of yolk content due to damage (arrow)

Differences between the *F. hepatica* eggs in the EAEAL ovicidal test group and the eggs in the control group were detected. On Day 1 of the test, prior to incubation, normal morphology of the eggs was observed, and the zygote and yolk were identified (Fig. 2A). On Day 7 of incubation, normal development of the zygote was observed in the control groups (Fig. 2B), whereas in the groups treated with EAEAL a slight change in the periphery of the shell was detected, suggesting structural damage that prevents proper larval formation (Fig. 2C). After 16 days of incubation, the untreated controls exhibited complete development of the miracidia (Fig. 2D) and were released after exposure to the photoperiod, leaving the shells empty (Fig. 2E). On the other hand, in the eggs treated with EAEAL, damage to the shell and leakage of the yolk content were visualized; this appeared to have affected yolk integrity and prevented the development of blastomeres in some cases (Fig. 2F) and completion of organogenesis or release of miracidia in others (Fig. 2G).

**Fig. 3:**
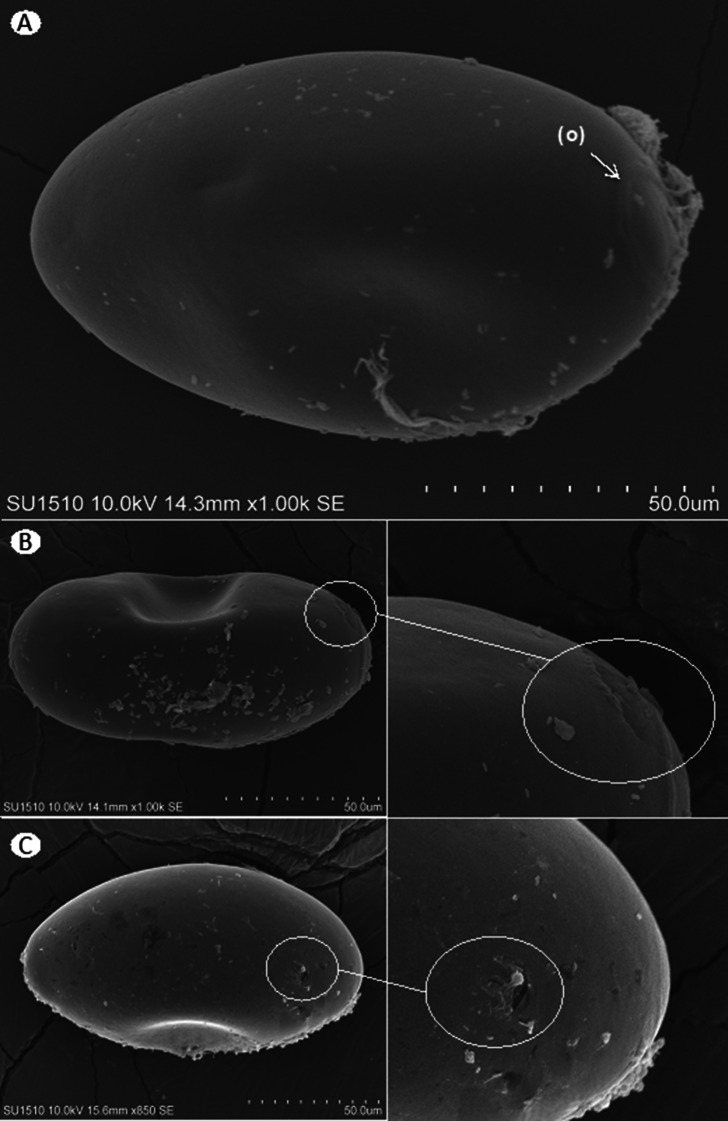
SEM of F. hepatica eggs from the EAEAL ovicidal test: (**A**) Control without treatment, the operculum (o) can be seen. (**B**) Treated with 100 mg/L EAEAL, damage is seen (circle). (**C**) Treated with 500 mg/L, damage is seen (circle)

The damage to the shell can be seen in greater detail in the images obtained via SEM. Compared with eggs treated with 100 mg/L EAEAL, control eggs did not exhibit damage to their shells (Fig. 3A), whereas in the treated eggs a small break in the shell and a concave region at its lower portion can be seen, suggesting escape of the yolk content to the outside (Fig. 3B). Eggs treated with 500 mg/L EAEAL showed greater rupture and more pronounced concavity (Fig. 3C).

### Fasciolicidal Activity

All control groups without treatment in the fasciolicidal bioassays maintained excellent mobility and did not present alterations during the evaluation. The fasciolicidal efficacies of EAEAL are shown in Table 1, obtaining efficiencies similar to those described by Ezeta et al. (2024). Probit analysis showed that the lethal concentration (LC)_50,_ LC_90_ and LC_99_ values from EAEAL with efficacy in adults determined in this experiment are 54.8 mg/L, 265.3 mg/L and 312.7 mg/L, respectively.

**Table 1:** Fasciolicidal percentages of EAL, TCBZ and ART, by evaluation times

Hours		Concentration mg/L						
	Control	ART		TCBZ	EAEAL			
		10	20	10	125	250	375	500
24	0	100	100	100	82 ± 0.071	98 ± 0.065	100	100
48	0	100	100	100	98 ± 0.065	100	100	100

(*p* < 0.05). Control: No treatment; ART: Artemisinin; TCBZ: Triclabendazole; EAEAL: Ethyl Acetate Extract *Artemisia ludoviciana;* ±: Standard deviation

### SEM Observations and Histology

**Fig. 4:**
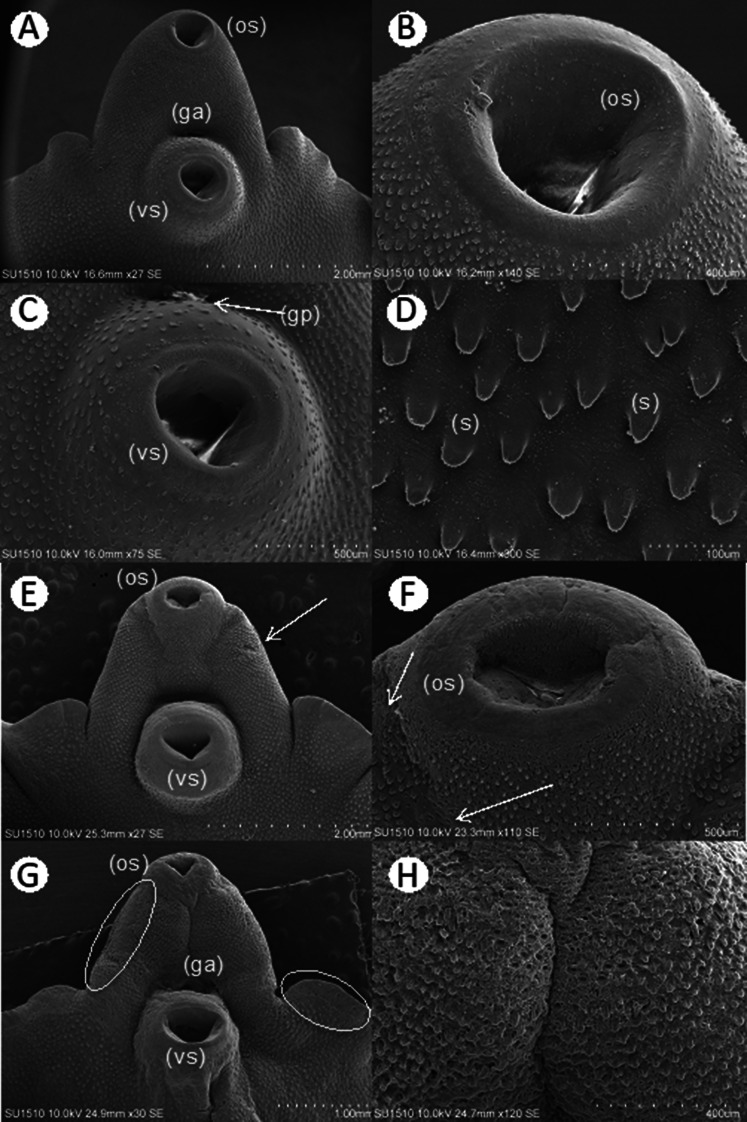
SEM of Fasciola hepatica: (**A**) Oral sucker (os), ventral sucker (vs.) and genital atrium (ga) at 48 h in a specimen without treatment. (**B**) Oral sucker (os) at 48 h in a specimen without treatment. (**C**) Ventral sucker (vs.) and genital pore (gp) at 48 h in a specimen without treatment. (**D**) Spines (s) of the tegument at 48 h in a specimen without treatment. (**E**) Treatment with 10 mg TCBZ damaged the tegument (arrow). (**F**) Oral sucker (os) treated with 10 mg of TCBZ, with erosion of spines (arrow) at 24 h postexposure. (**G**) At 24 h after treatment with 10 mg of ART, detachment of the tegument (circles) was observed. (**H**) At 24 h after treatment with 10 mg of ART, the tegument and spines were eroded

SEM revealed no alterations in the integument or its structures in the control groups (Fig. 4, A-D) compared to those in the treated groups. The group treated with TCBZ showed changes in the integument (Fig. 4E, arrow), erosion of the spines in certain areas (Fig. 4F, arrow) and thickening of the oral and ventral suction cups (Fig. 4E and F). The groups treated with ART exhibited damage to the integument and detachment of the integument in some regions (Fig. 4G, circles), exposing the muscle layers. Damage to and erosion of the spines was evident (Fig. 4H).

**Fig. 5:**
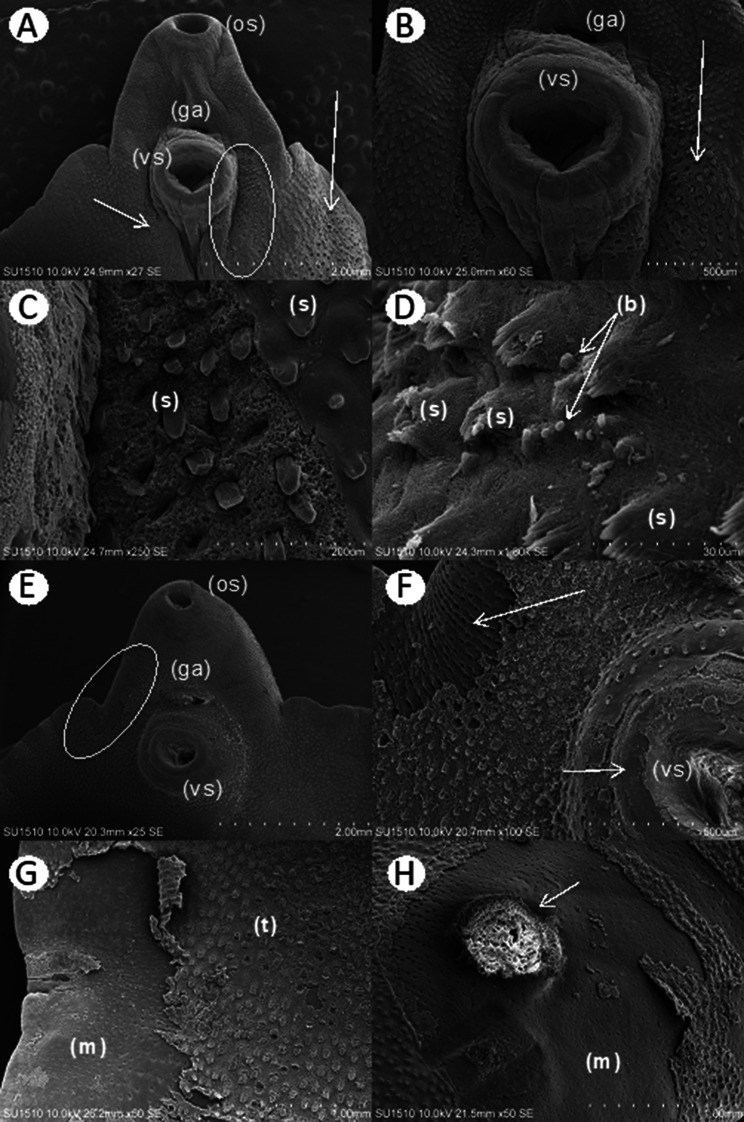
SEM of Fasciola hepatica exposed to EAEAL. **A**-**D**: Specimens treated with EAEAL at 125 mg/L. (**A**) Erosion in spines and tegument in different areas (circle and arrow); the oral sucker (os), the ventral sucker (vs.) and the genital atrium (ga) can be seen. (**B**) Close-up image of the periphery of the ventral sucker (vs.) showing damage (arrow). (**C**) Erosion of the tegument and spines (s). (**D**) Damage to spines (s) with vesicle formation (b). E-H: Specimens treated with EAEAL at 500 mg/L. (**E**) Detachment of the tegument (circled); the oral sucker (os), ventral sucker (vs.) and genital atrium (ga) can be seen. (**F**) Detachment of the tegument with exposure of muscle fibers in the lateral area and in the region of the ventral sucker (vs.) (arrows). (**G**) Erosion and detachment of the tegument (t) exposing the muscular layer (m). (**H**) Tegument detachment, showing severe damage in the muscular layer (m)(arrow)

Specimens exposed to 125 mg/L EAEAL exhibited partial erosion of the integument in different regions (Fig. 5A, circle and arrow). When the region of the oral sucker was observed at higher magnification, damage to the tegument that affected the integrity of the spines was seen (Fig. 5B, arrow and Fig. 5C). In some specimens exposed to the same concentration of EAEAL, small vesicles were observed around the spines (Fig. 5D, arrows), and when examined in detail, the changes in their morphology could be distinguished. In parasites treated with 500 mg/L EAEAL, similar but more pronounced damage that caused total detachment of the tegument (Fig. 5E, circle; Fig. 5F, arrows; Fig. 5G and H), exposure of the muscle layers and, in some cases, severe damage to the muscles (Fig. 5H, arrow) was observed.

**Fig. 6:**
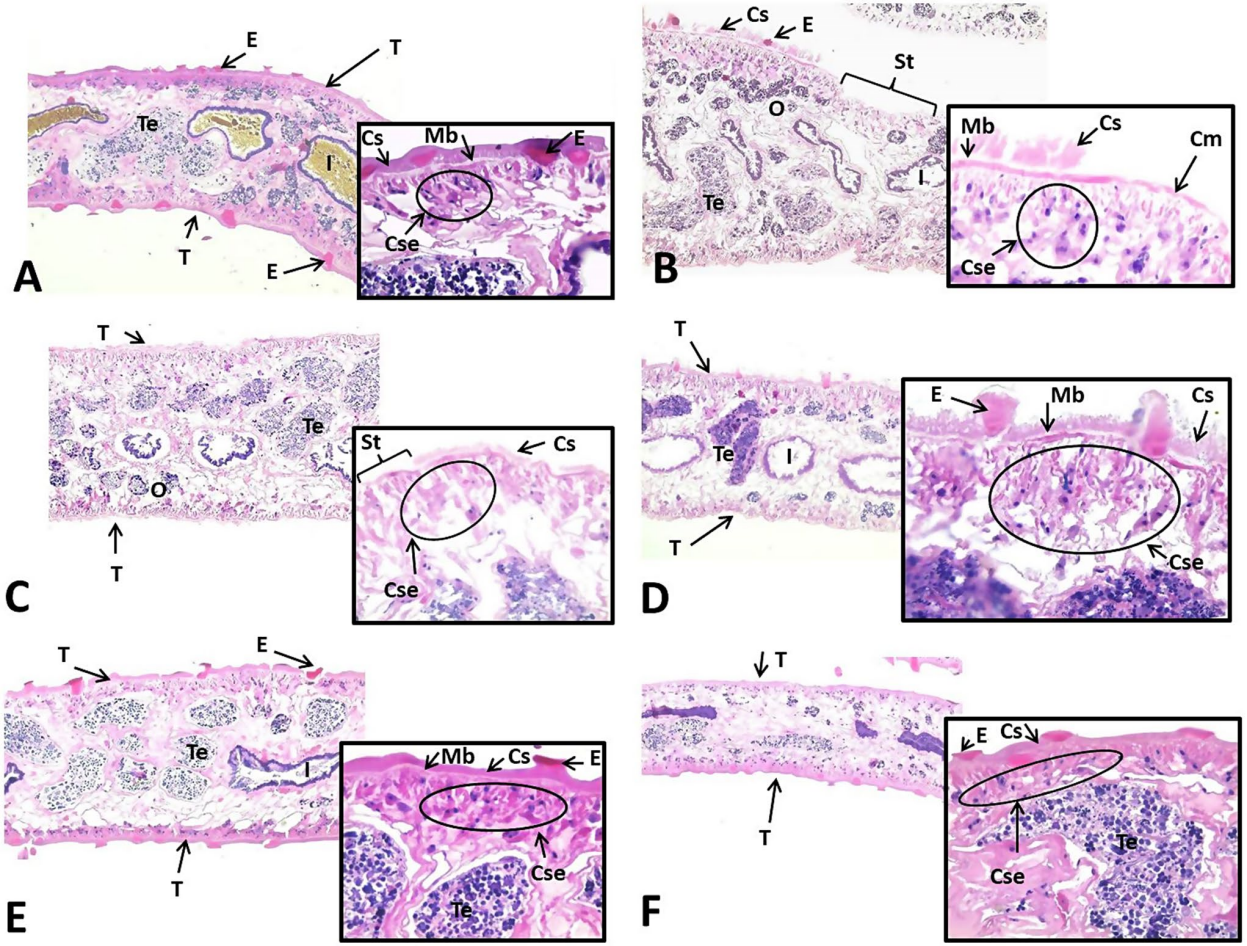
F. hepatica body sections from the different treatment groups. (**A**) Control: Intact, normal tegument is observed. The surface of the syncytial layer is smooth with unexposed spines. Below, a continuous basal membrane and normal subepidermal cells can be distinguished. (**B**) TCBZ 10 mg: An area in which the syncytial layer, the basal membrane, and even part of the muscle layers have been lost can be observed. In the area in which the tegument remains, the syncytial layer is altered and presents deformed spines. The altered subepidermal cells are shown in the inset. (**C**) 100 mg/L EAEAL: A tegument with an altered and discontinuous syncytial layer, in which the basal membrane is exposed in some areas, is seen. There is an almost complete absence of spines. In the inset, hypertrophic, vacuolated, and degenerative-appearing subepidermal cells are shown. (**D**) EAEAL 500 mg/L: A reduction in the thickness of the syncytial layer is observed, and there are abnormal filiform projections and deformed spines. The inset more clearly shows the changes in the syncytial layer, which include hypertrophy and vacuolization of the subtegumentary cells. (**E**) ART 10 mg: The tegument appears nearly normal, with only slight alterations in the shape of the spines. The inset shows the normal tegument and subtegument. (**F**) ART 20 mg: The tegument on both sides of the organism appears intact, but on one side, the spines have been lost. The inset shows a practically normal tegument and subtegument. H-E staining, 100× magnification (insets 400×) T: tegument, E: spines, I: intestine, O: ovary, Te: testicle, Cse: subepidermal cells, Mb: basal membrane, Cm: muscular layer, St: without tegument

In the parasites in the control group, the tegument showed histological homogeneity. In these specimens, the tegument is formed by a homogeneous syncytial layer with areas of clear striations. Spines, which may be everted, uneverted, or in formation, are located in this layer. Below the syncytial layer lies the basement membrane. In the subtegumental zone, normal muscle fibers were observed. Uninucleated subtegumentary cells exhibit diverse shapes and may infiltrate the muscle. Further inside the body, a meshwork of fibers and extracellular matrix is identified. The most recognizable organs are the ovary, the testicle, and the intestine (Fig. 6A). In the group treated with TCBZ, the tegument presented severe alterations. The syncytial layer had disappeared in many regions, leaving the basement membrane exposed. In areas in which the syncytial layer remained, the smooth surface of the layer was lost, and it appeared ragged. Spines were scarce and exhibited irregular and abnormal shapes; in some regions, the basement membrane had also disappeared. The layers of muscle cells showed no apparent pathological changes. In the subtegumental zone, cells were scarce, hypertrophic, and exhibited abundant cytoplasmic vacuolations. At the intestinal level, necrotic epithelium was observed, characterized by karyolysis and loss of laminar organization; in some areas, the epithelium was complete absent. In the ovary, many oocytes exhibited abnormal vacuolations. Changes in the testicle included disintegration of the cystic groupings of germ cells and likely alterations in meiosis (Fig. 6B).

The tegument of individuals treated with 125 mg/L EAEAL showed multifocal alterations. The affected tegument was characterized by the nearly complete absence of a syncytial layer, leaving many sites at which the basement membrane was exposed. In areas where the syncytial layer remained, abnormal outward filiform projections were observed. The spines were flat and had lost their characteristic eosinophilia. Many sites on the tegument lacked spines. The basal membrane showed changes in thickness, and in some parts, its absence exposed the muscular layers and caused degenerative changes. A significant decrease in the population of subepidermal cells in the subtegumentary region had resulted in large intercellular spaces. Some cells were dispersed and showed degenerative and necrotic changes; others were hypertrophic and showed significant changes in shape, and still others exhibited cytoplasmic vacuolations. The intestinal epithelium appeared necrotic. In the ovary, dead oocytes were identified, and there was disruption of the cystic arrangement of the testicle (Fig. 6C). The tegument of individuals treated with 500 mg/L EAEAL showed a heterogeneous reaction. It presented a patchy appearance, alternating between regions with moderate alterations and areas with severe alterations. In the most affected areas, the syncytial layer was absent, leaving the basal membrane exposed. In regions where the syncytial layer remained, it was altered and had developed abundant filiform projections. The spines could be atrophic, hypertrophic, or deformed. The basal membrane was discontinuous and variable in thickness. The muscular layers were atrophic and in some cases had disappeared. In the subepidermal region, there was a decrease in the number of cells; the remaining cells presented degenerative and necrotic changes, and many contained abundant vacuoles. The changes in the ovary included necrosis of oocytes, leading to a decrease in the number of oocytes in the ovarian follicles, which were depopulated or empty. In ovarian follicles containing dead oocytes, an external reaction of parenchymal cells with a necrotic appearance was observed. At the testicular level, changes consisted of disruption of the spermatogenic differentiation sequence, with rupture of the cysts of germ cells and the presence of cellular debris inside the testicular follicles (Fig. 6D).

In the specimens treated with ART at 10 mg/L, the basal membrane appeared continuous and of normal thickness. The subtegumentary layers were well preserved; the subtegumentary cells appeared slightly dispersed and showed mild changes in shape, and some contained vacuoles. Focal necrosis was observed in the intestine, while the spermatogenic sequence in the testicle did not appear to be altered. The ovaries appeared generally normal, although vacuolated oocytes were detected (Fig. 6E). Treatment with 20 mg/L ART resulted in mild changes in the tegument. Although the syncytial layer was intact with a smooth surface and normal thickness, slight vacuolization was noted, and some of the spines were deformed. The basal membrane appeared normal, as did the subtegumentary muscle layers. The most significant changes were observed in the subepidermal cells, which were scarce or absent in some areas and exhibited vacuolization. Focal epithelial necrosis was observed in the intestine. In the testicle, normal differentiation sequences were observed, and the supporting somatic cells exhibited mild vacuolization. In the ovary, some abnormally vacuolated oocytes were identified (Fig. 6F).

## Discussion

EAEAL has shown a promising fasciolicidal effect on adult fasciolae, generating significant damage to the parasite’s tegument and obtaining efficiencies similar to those described by Ezeta et al. (2024). In a previous study, this effect was observed in recently excysted fasciolae, which showed significant damage to their integument [22]. However, it was necessary to evaluate the response of adult specimens of the parasite to the extract because of the changes and development that the parasite undergoes over the course of several weeks. It is important to remember that, until the discovery of TCBZ, the drugs that have been used to treat fasciolosis do not affect all life stages of *F. hepatica*; this is why it is important to perform bioassays using different life stages of the parasite [45].

In a previous work, Ezeta and collaborators (2020) [22] verified the presence of artemisinin as a metabolite in an EtAc extract of *A. ludoviciana* Nutt. spp. mexicana and the metabolite that is most likely to be responsible for the fasciolicidal effect of the extract in recently excysted specimens. Artemisinin is a metabolite that is consistently present within the genus *Artemisia* and is an important drug in the treatment of malaria [46, 47]. Over the years, semisynthetic derivatives of ART have shown in vitro anticancer, anti-inflammatory, antimicrobial, antiviral, and antiparasitic effects, among others [48–50].

There are several current hypotheses about the mechanism of action of ART and its derivatives in trematodes. An important characteristic of ART and its derivatives is the presence of an endoperoxide bridge that interacts with the heme group in blood and generates a reduction reaction with the iron present, triggering the release of free radicals that cause alkylation of proteins and/or cellular lipids within the parasite. ART derivatives also interrupt the activity of the sodium-potassium pump (Na+/K+-ATPase) of the cell membranes in the *F. hepatica* tegument. Another possible mechanism of action of ART is changes in membrane permeability that cause an imbalance in mitochondrial functions, inhibiting oxidative phosphorylation and the transport chain and disrupting energy metabolism. Furthermore, it has been suggested that mitochondrial changes and the presence of lysosomal enzymes activate apoptotic mechanisms in parasite cells [51–53].

Histological evaluation of each individual specimen at different body levels allowed us to observe areas with no apparent pathological changes alternating with areas of advanced deterioration and regions with intermediate changes. This observation was consistent in almost all organs visible in transverse sections. McConville et al. (2009) [54] also reported that while use of the fasciolicide TCBZ results in damage to the tegument, there are other areas that appear normal. McKinstry et al. (2003) [55] suggested that extrategumentary effects are possible, given that the tegument is the first line of defense. Alterations at this level allow substances access to deep organs, which can then be damaged. In this study, EAEAL affected virtually the entire interior of the organism, including the digestive tract, the gonads, the internal parenchyma, and the subepidermal cells.

EAEAL has extrategumentary effects and appears to affect subtegumentary cells, which exhibited degenerative and necrotic changes at both concentrations tested. These observations, together with the proximity of these cells to the integument, suggest that the subtegumentary cells may play a role in the formation and maintenance of the integument since the syncytial layer and denticles must undergo a continuous formation process. In the group treated with the extract, the alterations found at the gonadal level, particularly at the 500 mg/L, are similar to those described after TCBZ treatment by Hanna et al. (2015) [12], Hanna et al. (2012) [43], and after closantel treatment by Scarcella and collaborators (2016) [44]. On the other hand, in previous work with semisynthetic derivatives of artemisinin (artesunate and artemether), which cause reproductive damage and alterations in the tegument of *F. hepatica*, an interruption in spermatogenesis in adult fasciolae affecting the reproductive system was observed [56, 57].

All specimens in the groups treated with EAEAL at any of the tested concentrations presented greater deterioration of the integument than did those in the groups treated with ART. In the latter, minor integumentary changes and focal or multifocal damage were identified, and these alterations could explain the mild changes observed in the intestine, ovary and testicle. It is important to consider that EAEAL, an extract, contains a wide variety of metabolites, and although the families of compounds present in it have previously been identified [22], it is important to continue research on the compounds with fasciolicidal effects.

In summary, the results of this work demonstrate that EAEAL is as effective as the fasciolicides that are currently widely used: it induces nearly all the histopathological changes in the integument described thus far. One of the most notable changes brought about by EAEAL is its effect on subtegumentary cells, an effect that should be studied further, as well as its effects on multiple organs, which highlight the complexity of the interaction between the parasite and EAEAL. The tegument functions in the absorption and exchange of nutrients, ionic regulation, and protection against the host’s immune reaction [58]. The importance of considering the damage caused to the reproductive system of *F. hepatica* lies in the fact that an adult that has successfully lodged in the bile duct of a host can shed between 20,000 and 50,000 eggs per day into the environment, influencing the epidemiological range of the parasite [2].

It is important to highlight that the extract had a greater effect on adult specimens due to the synergy of the compounds contained in it. Previously, Ezeta and collaborators (2020) [22] identified sesquiterpene lactones and artemisinin within the extract. Through fractionation and in vitro evaluation, these compounds were revealed as the possible agents responsible for the fasciolicidal effect, in that instance with recently excysted fasciolas. Therefore, it is necessary to continue elucidating the exact compounds.

Eggs treated with EAEAL showed damage to the surface of the shell, compromising internal stability, delaying larval development and, in some cases, inhibiting it completely, unlike the negative control groups, in which normal hatching occurred. The observed damage caused a considerable decrease in the hatching rate of the miracidia, as indicated by the ovicidal activity of EAEAL. Unfortunately, there are no concrete data on the mechanism of action of ART and its derivatives on trematode eggs, much less on the mechanism of action of EAEAL, to date; for this reason, it is necessary to continue relevant research that delves more deeply into this topic.

The findings reported here provide a foundation for future research on the exact identification and the mechanism of action of the secondary metabolites involved in the effects of EAEAL on *F. hepatica* and provide an opportunity to optimize the use of natural products in the comprehensive control of fascioliasis.

## Conclusion

Exposure of adult *Fasciola hepatica* to an ethyl acetate extract of the leaves of *Artemisia ludoviciana* Nutt. spp. *mexicana* causes damage to the tegument, intestine, ovaries and testes of the animals. In addition, it damages the eggshells of these parasites.

## Data Availability

No datasets were generated or analysed during the current study.
